# Frequency of macrolide resistance-associated mutations in *Mycoplasma genitalium*: a multicenter molecular analysis based in Brazil

**DOI:** 10.1590/S1678-9946202668070

**Published:** 2026-10-19

**Authors:** Fernando Hartmann Barazzetti, Marcos André Schörner, Vilmar Benetti, Henrique Borges da Silva Grisard, Julia Kinetz Wachter, Jéssica Motta Martins, Christinni Machado Venturi, Pâmela Cristina Gaspar, Angélica Espinosa Miranda, Dráurio Barreira, Glauber Wagner, Maria Luiza Bazzo

**Affiliations:** 1 Universidade Federal de Santa Catarina, Centro de Ciências da Saúde, Laboratório de Biologia Molecular, Microbiologia e Sorologia, Florianópolis, Santa Catarina, Brazil; 2 Universidade Federal de Santa Catarina, Programa de Pós-Graduação em Biotecnologia e Biociências, Laboratório de Bioinformática, Florianópolis, Santa Catarina, Brazil; 3 Universidade Federal de Santa Catarina, Programa de Pós-Graduação em Biotecnologia e Biociências, Laboratório de Virologia Aplicada, Florianópolis, Santa Catarina, Brazil; 4 Universidade Federal de Santa Catarina, Programa de Pós-Graduação em Farmácia, Laboratório de Biologia Molecular, Microbiologia e Sorologia, Florianópolis, Santa Catarina, Brazil; 5 Ministério da Saúde, Departamento de HIV/AIDS, Tuberculose, Hepatites Virais e Infecções Sexualmente Transmissíveis, Brasília, Distrito Federal, Brazil; 6 Universidade Federal do Espírito Santo, Programa de Pós-Graduação em Doenças Infecciosas, Vitória, Espírito Santo, Brazil

**Keywords:** Mycoplasma genitalium, Macrolide resistance, Quinolone resistance, Public health.

## Abstract

*Mycoplasma genitalium* is an emerging sexually transmitted pathogen with increasing antimicrobial resistance worldwide. In Brazil, molecular testing for *M. genitalium* and resistance-associated mutations is not routinely available, and national data remain scarce. We analyzed 118 *M. genitalium*-positive urethral and vaginal swab samples collected between 2018 and 2023 from sites across all five Brazilian geographic regions. PCR amplification and sequencing of *23S rRNA*, *parC*, and *gyrA* identified macrolide and fluoroquinolone resistance-associated mutations. Among 79 samples successfully sequenced for *23S rRNA*, 40.5% harbored macrolide resistance-associated mutations (A2058G, A2059G, or A2058T) detected in samples from all five regions at different frequencies. A total of 6.1% of sequenced samples presented *parC* mutations, including two with combined *23S rRNA* and *parC* mutations. No resistance-associated mutations were detected in *gyrA*. While resistance-associated mutations occurred in both symptomatic men and pregnant women, these findings are not representative estimates for the Brazilian population since the study used convenience samples from two distinct surveillance populations. Nonetheless, they provide multicenter molecular data that may inform future surveillance, diagnostic strategies, antimicrobial stewardship, and resistance-guided management of *M. genitalium* in Brazil.

## INTRODUCTION

The World Health Organization (WHO) estimates that approximately 357 million new cases of curable sexually transmitted infections (STI) occur annually worldwide; however, infections caused by *Mycoplasma genitalium* (MG) are not included in these global estimates^1^ ^),(^ ^2^. Though the *M. genitalium* infection prevalence varies from 1% in the general population to over 9% in higher-risk groups, the situation is aggravated by the high prevalence of antimicrobial resistance, which reaches 33.3% for macrolides globally and exceeds 60% in specific urban centers^3^ ^-^ ^5^. Despite its recognized role in urogenital infections, epidemiological data on MG remain limited, particularly in low- and middle-income countries, raising concern about its actual public health burden^6^ ^),(^ ^7^.

MG infection is frequently asymptomatic in both men and women, contributing to sustained transmission^8^ ^),(^ ^9^. In men, MG is a well-established cause of nongonococcal urethritis (NGU) and may be associated with complications such as epididymitis and reactive arthritis^9^. In women, infection has been linked to cervicitis, pelvic inflammatory disease, adverse pregnancy outcomes, and tubal infertility, although clinical manifestations may be absent in up to 40%-75% of cases^9^ ^),(^ ^10^ ^),(^ ^11^. Substantial proportion of asymptomatic infections reinforces the importance of surveillance strategies beyond symptom-based management.

 The increasing prevalence of antimicrobial resistance (AMR) in MG represents a major challenge for STI control programs worldwide. Resistance is primarily mediated by single nucleotide polymorphisms (SNPs) in the 23S rRNA gene, associated with macrolide resistance, and in the *parC* and *gyrA* genes, associated with fluoroquinolone resistance^12^ ^),(^ ^13^. Mutations at nucleotide positions 2058 and 2059 (Escherichia coli numbering) in the 23S rRNA gene, and amino acid substitutions at positions S83 and D87 in *parC*, are the most reported resistance-associated mutations^12^. Systematic reviews and meta-analyses reveal a rapid global increase in macrolide resistance, approaching or exceeding 50% in several regions^3^ ^),(^ ^5^ ^),(^ ^7^, prompting international guidelines to recommend resistance-guided therapy when molecular testing is available.

In Brazil, STI management within the Unified Health System (Sistema Unico de Saude - SUS) is largely based on syndromic approaches. For NGU, azithromycin 1g in a single dose remains the recommended first-line therapy, with doxycycline as an alternative option^14^ ^),(^ ^15^. When MG is identified, azithromycin continues to be recommended and well-defined second-line options are limited^14^. However, molecular diagnostic testing for MG and detection of resistance-associated mutations are not routinely available in the public health system. As such, empirical treatment without resistance profiling may contribute to selective antimicrobial pressure and further dissemination of resistant strains. In middle-income countries where syndromic management predominates, surveillance of MG resistance-associated mutations is particularly critical to prevent large-scale dissemination of untreatable infections^16^.

Although molecular diagnostic capacity has expanded in Brazil since 2017-2018^17^ ^-^ ^19^, national data on MG resistance remain scarce and fragmented. Given the reliance on syndromic management and the growing international evidence of AMR, generating nationwide surveillance data is essential to update clinical guidelines and strengthen antimicrobial stewardship policies. To date, no nationwide molecular surveillance study has evaluated MG resistance-associated mutations across all Brazilian regions, limiting the evidence base required for policy revision. Thus, this study evaluated macrolide- and fluoroquinolone-associated resistance mutations in MG-positive clinical samples collected across all five Brazilian geographic regions, providing molecular evidence to support national STI treatment policies.

### Ethics

This study integrates the Brazilian surveillance of *Neisseria gonorrhoeae* antimicrobial susceptibility and Brazilian Surveillance for STIs in Pregnant Women, approved by the Committee of Ethics in Research with Human Beings under CAAE 83053818.4.0000.0121 and CAAE 52622421.5.0000.0121, respectively.

## MATERIALS AND METHODS

### M. genitalium-positive clinical specimens

A convenience sample of *M. genitalium*-positive specimens was selected from two previously conducted multicenter surveillance studies. Samples were included irrespective of the original epidemiological objectives of those studies, as the present analysis focused exclusively on molecular characterization of resistance-associated mutations. A total of 118 individual clinical samples positive for *M. genitalium* were included in the study, 50 from male urethral discharge (detected by Allplex TM CT/NG/MG/TV Assay - Seegene^®^) and 68 from vaginal swabs of pregnant women (detected by Hologic Panther platform - San Diego, USA). These samples were obtained from a total of 3,917 samples provided by three studies^17^ ^),(^ ^20^, and collected between 2018 and 2023. Male urethral discharge samples were collected using a flocked swab (FLOQSwabs^®^- Copan) and stored in Universal Transport Media (UTM-RT^®^- Copan), whereas vaginal swabs were collected with Multitest Swab Specimen Collection Kit (Aptima^®^) and stored in Multitest Swab Transport Media (Aptima^®^). All samples originated from the five major geographic regions of Brazil and were collected predominantly in capital cities, metropolitan areas, and large urban centers rather than in small towns or rural settings. Participating sites included Belo Horizonte, Brasilia, Contagem, Curitiba, Florianopolis, Fortaleza, Guarulhos, Manaus, Pelotas, Porto Alegre, Recife, Ribeirao das Neves, Ribeirao Preto, Salvador, Sao Jose dos Campos, Sao Paulo, and Vitoria. Samples were transported in dry ice and upon arrival at the Laboratory of Molecular Biology, Microbiology and Serology (Federal University of Santa Catarina), where the study was conducted, they were stored at −80 °C. All samples included in this study were anonymized.

This analysis was not designed to estimate the prevalence of resistance-associated mutations in Brazil, but rather to characterize resistance-associated mutations among available *M. genitalium*-positive specimens obtained from two previously conducted multicenter surveillance studies.

## DNA purification and sequencing

Following manufacturer’s instructions, the genetic material was isolated from all samples using ReliaPrep^™^ Blood gDNA Miniprep System Kit (Promega^®^). For SNPs identification, firstly an amplification of *parC, gyrA* gene, and 23S rRNA of *M. genitalium* was performed following the protocol described by Pitt *et al*.^9^ using GoTaq^®^ Hot Start Polymerase (Promega^®^) amplification kit. DNA amplicon Sequencing was performed by ACTGene Análises Moleculares Ltd. (Center for Biotechnology, UFRGS, Porto Alegre, RS, Brazil) using BigDye Terminator v3.1 Cycle Sequencing Kit (Applied Biosystems^®^) alongside ABI 3500 Genetic Analyzer (Applied Biosystems^®^). Electropherograms of the obtained sequences were trimmed, excluding bases with Phred quality less than 20 on Chromas software (version v2.6.6, Technelysium Pty Ltd., South Brisbane, AUS), and aligned to *M. genitalium* reference strain G37 (GenBank: L43967.2) on MEGAX software (version v10.2, MEGA Team, Philadelphia, PA, EUA)^21^.

## Statistical analysis

Descriptive analyses were presented as absolute frequencies and proportions. No inferential comparisons were performed between geographic regions or source populations for the study was based on a convenience sample, with small and unequal numbers of successfully sequenced specimens across strata and was not designed or powered for between-group comparisons.

## RESULTS

### Study population and geographic distribution

The study included 118 *Mycoplasma genitalium*-positive clinical samples collected between 2018 and 2023 from all five Brazilian geographic regions. The South region contributed 30 (25.4%) samples, the Southeast region 48 (40.7%), the Central-West region 12 (10.2%), the Northeast region 21 (17.8%), and the North region 7 samples (Figure 1). Of the total, 50 were male urethral discharge specimens and 68 were vaginal swabs collected from pregnant women.

**Figure 1:**
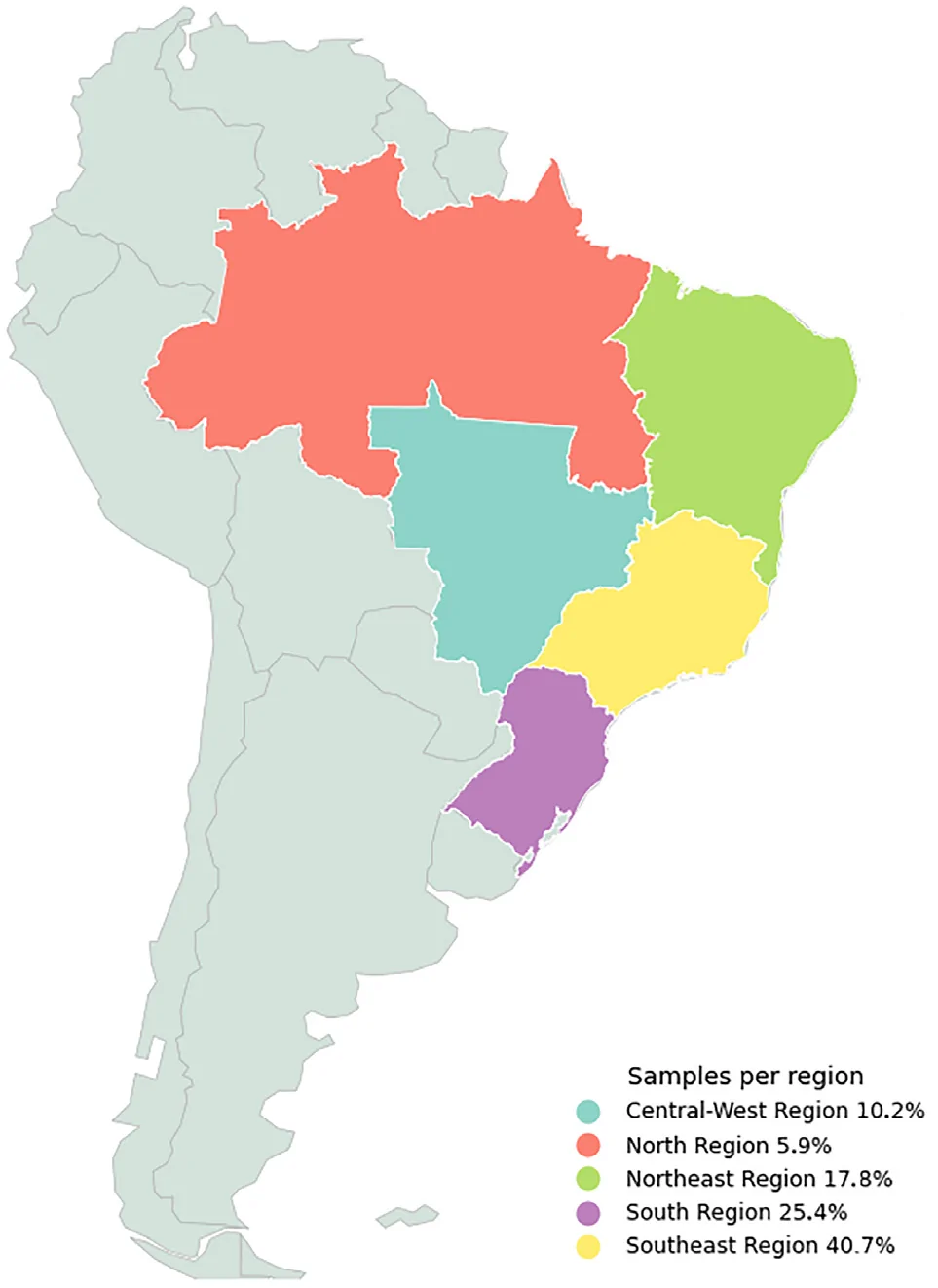
Number and swab type of samples from each Brazilian geographic region.

### Sequencing success and target amplification

Of the 118 MG-positive samples, 98 yielded high-quality sequences in at least one of the investigated targets, totaling 243 successfully sequenced gene targets: 79 for 23S rRNA, 82 for *parC*, and 82 for *gyrA*. Complete sequencing of all three targets was achieved in 42 samples. Source population (symptomatic men and pregnant women) and geographic region distribution was similar between successfully sequenced and non-sequenced samples, suggesting that sequencing failures were unlikely to introduce substantial selection bias regarding these characteristics.

### Macrolide resistance-associated mutations

Mutations in the 23S rRNA gene at positions 2058 and 2059 (*E. coli* numbering), associated with macrolide resistance, were detected in 40.5% (32/79) of successfully sequenced samples. Identified mutations included A2058G (14/79), A2059G (14/79), and A2058T (4/79).

Macrolide resistance-associated mutations were identified in samples from all five geographic regions: South (12/20), Southeast (10/27), Central-West (4/8), Northeast (5/17), and North (1/7). These mutations were observed in both male urethral discharge samples and vaginal swabs from pregnant women.

### Fluoroquinolone resistance-associated mutations

Mutations in the *parC* gene were detected in 6.1% (5/82) of samples. Identified polymorphisms included G248A (1/82) and G259T or G259A (4/82) (MG numbering). Two samples presented resistance-associated mutations in both 23S rRNA (A2058G) and *parC* (G259T or G248A). No resistance-associated mutations were identified in the *gyrA* gene.

### Distribution of resistant genotypes

Resistance-associated mutations were detected throughout the study period (2018-2023) in both male urethral discharge samples and vaginal swabs from pregnant women. Figure 2 illustrates the distribution of single nucleotide polymorphisms (SNPs) according to collection year and specimen type. Two samples (BH42 and DF47) presented mutations in both 23S rRNA and *parC*, while other samples presented isolated *parC* or 23S rRNA mutations.

### International comparison of resistance frequency

To contextualize the national findings, the identified frequency of resistance-associated mutations was compared with data reported in selected international studies (Table 1). The 40.5% frequency of macrolide resistance-associated mutations among successfully sequenced samples in Brazil is consistent with rates reported in North America, Europe, and Australia, where resistance often exceeds 40%^3^ ^),(^ ^7^ ^),(^ ^22^ ^-^ ^24^.

**Table 1:** Selected international studies reporting resistance-associated mutations in Mycoplasma genitalium.

Region	Study period	Population	Sample size (n)	Macrolide resistance (%)	Fluoroquinolone resistance (%)	Study
Australia	2015-2019	STI clinic	22,974	48%	9%	Chua *et al*.^3^
USA	2017-2018	Multicenter	3,332	44%	5%	Gnanadurai *et al*.^25^
Europe (multicenter)	2010-2016	Mixed	8,508	40-60%	5-15%	Mateo Léon *et al*.^7^
South Africa	2015-2018	STI patients	4,636	15%	6%	Manhart *et al.* ^29^
Brazil	2018-2023	National multicenter	3,917	40.5%	6.1% (*parC*)	This study

**Figure 2:**
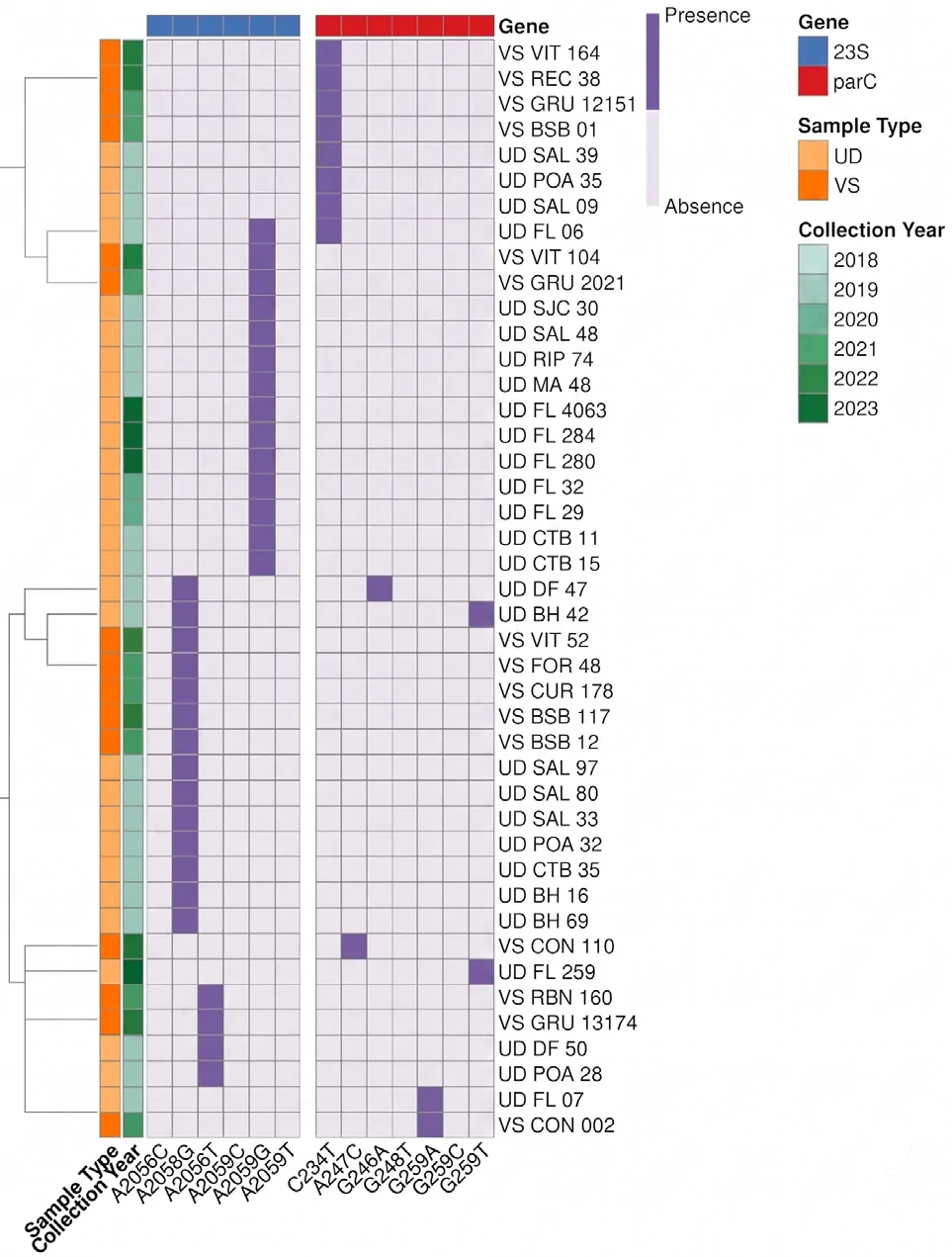
Hierarchical clustering heatmap of *Mycoplasma genitalium* samples (2018-2023) reveals three main resistance clusters: a top cluster with *parC* C234T and combined *23S* A2059G resistance (2022-2023); a central cluster dominated by isolated *23S* A2058G; and a smaller, recent bottom cluster (2022-2023) with *23S* A2058T and *parC* G259A. Left-side colored bars show sample type and collection year; the right purple matrix displays mutation presence; UD = urethral discharge; VS = vaginal swab.

Fluoroquinolone resistance-associated mutations in *parC* (6.1%) fell within the range described in several international settings, although lower than those observed in some European cohorts^4^ ^),(^ ^22^. The observed frequency of resistance-associated mutations among the sequenced samples was within the range reported in several international studies. However, direct comparisons should be interpreted cautiously due to differences in study design, populations, diagnostic assays, and resistance detection methods (Table 1).

## DISCUSSION

This multicenter molecular analysis identified a high frequency of macrolide resistance-associated mutations among successfully sequenced *M. genitalium*-positive specimens collected from different Brazilian geographic regions. Macrolide resistance-associated mutations occurred in samples originating from all five geographic regions albeit at different frequencies. The frequency observed among sequenced samples fell within the range reported in studies from North America, Europe, and Australia, where macrolide resistance-associated mutations are often detected at high levels^3^ ^),(^ ^7^ ^),(^ ^23^ ^-^ ^25^. However, direct comparisons should be interpreted cautiously due to differences in study design, populations, diagnostic assays, and resistance detection methods.

Fluoroquinolone resistance-associated mutations in *parC* occurred in a smaller proportion of samples (6.1%), consistent with rates reported in several international settings^7^ ^),(^ ^23^ ^),(^ ^26^. Importantly, two samples presented genotypes harboring mutations associated with both macrolide and fluoroquinolone resistance. Although no *gyrA* mutations were identified, continued surveillance remains important as combined mutations in *parC* and *gyrA* have been associated with treatment failure in other settings^11^ ^),(^ ^27^ ^),(^ ^28^. Detection of dual resistance markers, even at low frequency, signals a potential future threat if selective antimicrobial pressure persists.

These findings have important implications for STI management in Brazil. Within the SUS, nongonococcal urethritis management relies predominantly on syndromic approaches, with azithromycin-based regimens recommended as first-line therapy^14^ ^),(^ ^15^. Several international guidelines no longer recommend empirical single-dose azithromycin for *M. genitalium* infection due to the high frequency of macrolide resistance-associated mutations and documented treatment failure, favoring resistance-guided therapeutic approaches whenever molecular testing is available^3^ ^),(^ ^7^. In the absence of routine molecular testing for MG and resistance-associated mutations, empirical azithromycin use may contribute to selective antimicrobial pressure and therapeutic failure. This issue may be particularly relevant in middle-income settings where syndromic management remains the predominant approach and molecular diagnostic capacity is limited^7^ ^),^ ^26^. Molecular diagnosis of *M. genitalium* in symptomatic individuals and in cases of persistent or recurrent urethritis may contribute to more appropriate treatment decisions, in accordance with current international recommendations^3^ ^),(^ ^7^.

Brazil has successfully implemented national surveillance programs for *Neisseria gonorrhoeae*, showing the feasibility of coordinated antimicrobial resistance monitoring within the SUS framework. Laboratory infrastructure, sentinel site networks, and governance mechanisms established by these initiatives provide a practical foundation upon which molecular surveillance of *Mycoplasma genitalium* could be integrated into existing national platforms.

Another important finding was the detection of resistance-associated mutations in samples collected from pregnant women. Though MG screening is not routinely recommended during prenatal care, infection has been associated with adverse reproductive outcomes^9^ ^),(^ ^10^. Detection of resistant-associated mutations in this population highlights the need for further investigation regarding the epidemiology, clinical significance, and management of *M. genitalium* infection during pregnancy.

### Limitations

This study has limitations. First, the study population was derived from two distinct surveillance studies, including symptomatic men with urethral discharge and pregnant women attending antenatal care. Thus, our findings cannot be interpreted as estimates of the frequency of resistance-associated mutations in the Brazilian population and may be influenced by selection bias related to the characteristics of the source populations. Moreover, no formal sample size calculation or random sampling strategy was used for the present analysis, as the study was based on a convenience sample of *M. genitalium*-positive specimens. Second, not all MG-positive samples yielded high-quality sequences, likely due to low bacterial load in clinical specimens, a common challenge in MG molecular studies. However, the distribution of source population and geographic region was similar between successfully sequenced and non-sequenced samples, suggesting that sequencing failures were not strongly associated with these characteristics. Nevertheless, the possibility of residual selection bias cannot be completely excluded. Despite Nested PCR constituting one of the most widely recommended approaches for molecular characterization of this pathogen, successful sequencing remains challenging because of the limited quantity of available genetic material^29^ ^),(^ ^30^. Moreover, the heterogeneity of samples from different populations restricts in-depth epidemiological analysis and limits direct comparisons between groups. Finally, resistance-associated mutations were inferred exclusively from genotypic markers. Phenotypic susceptibility testing, previous antimicrobial exposure, and clinical treatment outcomes data were not available. Thus, our findings should not be interpreted as direct evidence of antimicrobial resistance or treatment failure. Rather, they provide molecular evidence on the presence of mutations previously associated with resistance in MG and contribute to understanding their distribution among the analyzed samples.

Despite said limitations, this study provides the first multicenter molecular overview of resistance-associated mutations in MG, including samples from all five Brazilian geographic regions. Since resistance-associated mutations were identified exclusively through genotypic analysis, and neither phenotypic susceptibility testing nor clinical outcome data were available, the findings should be interpreted as evidence of existing resistance-associated mutations rather than confirmed antimicrobial resistance or treatment failure. Detection of these mutations in samples collected from different geographic regions and population groups highlights the importance of continued molecular surveillance of *M. genitalium* in Brazil. Further studies using representative sampling strategies and incorporating clinical outcomes are needed to better inform treatment recommendations and public health policies.

## CONCLUSION

This multicenter molecular analysis detected macrolide resistance-associated mutations in *Mycoplasma genitalium* across samples from all five Brazilian geographic regions and from both symptomatic men and pregnant women. Given that resistance-associated mutations were identified exclusively via genotypic analysis, and the unavailability of both phenotypic susceptibility testing and clinical outcome data, these findings should be interpreted as evidence of existing resistance-associated mutations rather than confirmed antimicrobial resistance or treatment failure. Considering the convenience sampling strategy and heterogeneous source populations, these findings should not be considered representative estimates for the Brazilian population. Continued molecular surveillance and studies incorporating clinical outcomes are needed to better understand the epidemiology and clinical impact of resistance-associated mutations and to inform future diagnostic and therapeutic strategies for *M. genitalium* infection in Brazil.

## Data Availability

The Anonymized Dataset Generated During This Study Is Available From The Corresponding Author Upon Reasonable Request.
